# Secondary solid malignancies and precancerous lesions after allogeneic hematopoietic stem cell transplantation using non-total body irradiation-based conditioning in acute myeloid leukemia

**DOI:** 10.1007/s00432-024-05679-5

**Published:** 2024-03-22

**Authors:** Gruber Isabella, Appel Katharina, Edinger Matthias, Koelbl Oliver, Wolff Daniel

**Affiliations:** 1 Department of Radiation Oncology, University Hospital Regensburg, Regensburg, Germany; 2 Innsbruck Medical University, Innsbruck, Austria; 3 Department of Internal Medicine III, University Hospital Regensburg, Regensburg, Germany

**Keywords:** Allogeneic-hematopoietic stem cell transplantation, Secondary solid malignancies, Graft-versus-host disease, Chemotherapy-only conditioning, Acute myeloid leukemia

## Abstract

**Introduction:**

Long-term survivors have an increased risk of developing secondary solid malignancies (SSMs) after allogeneic-hematopoietic stem cell transplantation (allo-HSCT) with graft-versus-host disease (GVHD) potentially modulating these risks.

**Methods:**

This retrospective study analyzed the cumulative incidences of SSMs after chemotherapy-based conditioning for allo-HSCT patients with acute myeloid leukemia (n = 266) transplanted at the University Hospital Regensburg between 1999 and 2016.

**Results:**

The median follow-up was 11.4 years (Interquartile range, 9.0–14.9). The 100-day cumulative incidence of grade II-IV acute GVHD (aGVHD) was 44.4% [95% CI (38.3, 50.2)], while the 5-year cumulative incidence of chronic GVHD (cGVHD, requiring systemic immunosuppression) was 36.9% [95% CI (31.1, 42.6)]. The cumulative incidences of secondary squamous cell carcinomas (SCCs) at 10 and 15 years were 4.2% [95% CI (2.2, 7.2)] and 8.1% [95% CI (4.6, 12.8)], while the cumulative incidences of non-SCCs at 10 and 15 years were 5.4% [95% CI (3.1, 8.7)] and 6.9% [95% CI (4.0, 10.8)]. Antithymocyte globulin (ATG) was associated with reduced incidences of SCCs but not of non-SCCs. Patients with grade II-IV aGVHD had increased rates of SCCs after adjusting with patient age and ATG, while patients with cGVHD showed only a trend for increased rates of SCCs.

**Conclusion:**

The data indicate that aGVHD and cGVHD affect the rates of secondary SCCs. While the use of ATG is associated with lower incidences of SCCs via reduction of GVHD, there was no association of ATG with non-SCCs.

## Introduction

Allogeneic hematopoietic stem cell transplantation (allo-HSCT) is a curative treatment option for selected patients with acute myeloid leukemia (AML). However, allo-HSCT is associated with a relatively high long-term non-relapse mortality (NRM), including secondary solid malignancies (SSMs) (Curtis et al. 1997). Factors modifying the risks of SSMs are the primary diagnosis, genetic predisposition, patient age, conditioning regimens with either total body irradiation (TBI) or chemotherapy alone, infections with oncogenic viruses and graft-versus-host disease (GVHD) (Curtis et al. 1997; Miller and Johnstone 2001; Rizzo et al. 2009). Chronic GVHD (cGVHD) occurs in approximately 50% of patients and represents the main cause of long-term morbidity and mortality after allo-HSCT with acute GVHD (aGVHD) known to be the main risk factor (Lee et al. 2003; Stewart et al. 2004; Grube et al. 2016). It is still unclear how GVHD alters the risks of SSMs and whether non-squamous cell carcinomas (non-SCCs) and SCCs are similarly influenced by GVHD. Unfortunately, literature focusing on SSMs after allo-HSCT shows heterogeneity in conditioning regimens (TBI-based, chemotherapy-only), patient population (leukemia, lymphoma or aplastic anemia) and types of SSMs reported with little information on individual cancer types. In this retrospective study, we, therefore, analyzed the cumulative incidences of SSMs and precancerous lesions after non-TBI-based conditioning focusing on AML patients with the provision of details of all SSMs. The objective of this study was to analyze the effects of pre-transplantation variables on the cumulative incidences of SSMs and to estimate the effects of aGVHD and cGVHD on the rates for SSMs.

### Data collection

We retrospectively analyzed the cumulative incidences of SSMs, precancerous lesions (carcinomas in situ, actinic keratosis of the skin) and histologically proven atypical nevi after non-TBI-based conditioning in AML patients who received their 1st allo-HSCT at the Department of Hematology of the University Hospital Regensburg between 1999 and 2016. Post-transplant lymphoproliferative disorders were not analyzed. Secondary solid malignancies were subdivided into invasive squamous cell carcinomas (SCCs) and non-squamous cell carcinomas (non-SCCs). Eligibility criteria for this retrospective analysis included adult patients with primary or secondary AML (treatment-related AML) who underwent their 1st allo-HSCT from matched sibling donors (MSD), matched unrelated donors (MUD), mismatched unrelated donors (MMUD) or haploidentical/mismatched related donors (MMRD) after non-TBI-based conditioning. The choice of conditioning regimens and GVHD prophylaxis was based on the oncologists´ discretion and dependent on the patient´s age, disease risk, comorbidities and donor type (antithymocyte globulin was standard in unrelated donor transplantation and at the discretion of the physicians in sibling donor transplantation). As TBI-based patients are younger, we didn’t analyze TBI-patients to prevent an age bias. Clinical data were extracted from the medical charts of the Department of Hematology, University Hospital Regensburg. Transplantation variables included patient age at the time of allo-HSCT, sex, diagnosis, Karnofsky performance score (KPS), hematopoietic cell transplantation-comorbidity index (HCT-CI) as described by Sorror et al. (2005), 2017 European LeukemiaNet (ELN) genetic risk stratification as described by Döhner et al. (2017), disease status before allo-HSCT, stem cell source, conditioning regimens, recipient and donor characteristics (donor age, HLA-compatibility, gender match, cytomegalovirus serostatus), GVHD prophylaxis and the use of antithymocyte globulin (ATG). We captured grade II-IV aGVHD, cGVHD requiring systemic immunosuppressive therapy, the duration of systemic immunosuppressive therapy of cGVHD and sites of cGVHD (skin, oral mucosa, liver, lung, eyes, gastrointestinal tract, joints, fascia, genitals and cGVHD of the central nervous system). All patients received screenings for cutaneous malignancies before allo-HSCT. The screening program of SSMs after allo-HSCT included physical examinations including examinations of the skin, thyroid glands, oral cavity and pharynx during annual control. Patients at high risk for developing oropharyngeal cancer were screened every 6 months (e. g., in cases of GVHD). Colorectal, gynecological and urological screening was recommended once a year. Data closing was in October 2023. The local Ethics Board of the University of Regensburg approved this study (number, 20-1810-101).

### Definitions and statistical endpoints

The primary endpoints were the cumulative incidences of SSMs with deaths without prior SSMs considered as competing events. SSMs were subdivided into SCCs and non-SCCs. A separate analysis comprised precancerous lesions (carcinomas in situ, actinic keratosis of the skin) and histologically proven atypical nevi. For patients developing more than one SSM or precancerous lesions, the time to the first lesion was recorded. Acute GVHD was classified as clinically significant at grade II–IV aGVHD. Acute GVHD and cGVHD were defined according to described standard criteria (Filipovich et al. 2005; Jagasia et al. 2015). The cumulative incidences of grade II-IV aGVHD were estimated considering death or relapse without grade II-IV aGVHD as competing events. For the cumulative incidences of cGVHD requiring systemic immunosuppressive treatment, relapse or death without prior cGVHD (requiring systemic immunosuppressive treatment) was counted as a competing event. Additionally, we analyzed the effects of pre-transplantation variables on the incidences of SSMs (SCCs and non-SCCs) using a multivariable Fine-Gray regression model accounting for the respective competing events (death without prior SSM). Pre-transplantation covariates were patient age, conditioning regimens, donor type, graft source, sex match, donor age, donor/recipient CMV serostatus and the use of ATG. The impact of time-varying variables (aGVHD and cGVHD) on the rates of SSMs was analyzed using cause-specific hazard (CSH) models in a counting process format with adjustment of patient age and ATG. In cases with ongoing systemic immunosuppressive therapy at the time of diagnosis of cGVHD (e. g., therapy of aGVHD), we recorded the duration of the entire immunosuppressive therapy for GVHD. First-line treatment of cGVHD consisted of corticosteroids given alone or combined with calcineurin or mTOR inhibitors. The choice of second and third-line therapies depended mainly on the comorbidities, the risk profile of cGVHD and patients´ medical history. Information on the last day of systemic immunosuppressive therapy for cGVHD was missing in 6 patients with cGVHD. All times to the endpoints were calculated from the date of allo-HSCT (day 0). If a patient was event-free for all of the endpoints, the patient was censored at the last date of follow-up with confirmation of being event-free.

### Statistical analysis

Transplant-related characteristics are presented as median and interquartile range (IQR) for continuous variables and as absolute and relative frequencies for categorical variables. We used cumulative incidence functions (CIF) to describe the incidences of SSMs accounting for the competing risks (death without prior SSMs). Fine and Gray models described the effects of pre-transplantation variables on the subdistribution hazard functions. The proportional hazard assumption of the Fine and Gray models was tested by using Schoenfeld-type residuals. The effects of aGVHD and cGVHD on cause-specific hazards of SSMs were estimated with Cox proportional hazard regression treating all other events as censored. Acute GVHD and cGVHD were analyzed using a counting process format with adjustment of patient age and ATG. Hazard Ratio (HR) and 95% confidence intervals (95% CI) are presented as effect estimates. Median follow-up time was estimated using the reverse Kaplan–Meier method. All *P*-values were two-sided, and *P* < 0.05 were considered significant. Statistical analysis was performed using R, version 4.3.2 (R Core Team. R: A language for statistical computing. 2014. The R Foundation for Statistical Computing, Vienna, Austria) and SPSS 26.0 (SPSS Inc., Chicago, IL, USA).

## Results

### Patient and transplant characteristics

Table 1 summarizes transplant characteristics. Between 1999 and 2016, 266 patients received their 1st allo-HSCT for de novo/primary AML (n = 178) or secondary AML (n = 88) after non-TBI-based conditioning with peripheral blood (n = 244) or bone marrow (n = 22) as a stem cell source. The median patient age at allo-HSCT was 55.9 years (IQR, 45.8–61.4). The median follow-up time was 11.4 years (IQR, 9.0–14.9). All patients received reduced-intensity conditioning regimens (RIC-regimens) with Melphalan-based chemotherapy (n = 193) as the most frequent regimen. Table 2 summarizes all conditioning regimens.

**Table 1:** Patient and transplant characteristics in 266 patients

Characteristic	value
Follow-up, years, median (IQR)	11.4 (9.0–14.9)
Patient age at allogeneic hematopoietic stem cell transplantation, years, median (IQR)	55.9 (45.8–61.4)
Gender, n (%)	
Male	148 (55.6%)
Female	118 (44.4%)
Primary diagnosis, n (%)	
De novo, primary acute myeloid leukemia	178 (66.9%)
Secondary acute myeloid leukemia	88 (33.1%)
Karnofsky performance score	
< 80	43 (16.2%)
≥ 80	223 (83.8%)
Hematopoietic cell transplantation-comorbidity index (HCT-CI), n (%)	
0	71 (26.7%)
1–2	88 (33.1%)
≥ 3	107 (40.2%)
2017 ELN genetic risk stratification, n (%)	
Favorable	41 (15.4%)
Intermediate	112 (42.1%)
Adverse	113 (42.5%)
Disease status at 1st allo-HSCT, n (%)	
First complete remission, CR1	103 (38.7%)
CR2, first partial remission, PR1	77 (28.9%)
> CR2, refractory, active AML	86 (32.3%)
Donor type, n (%)	
Matched sibling donor	86 (32.3%)
Matched unrelated donor	124 (46.6%)
Mismatched unrelated donor	51 (19.2%)
Haploidentical, mismatched related donor	5 (1.9%)
Stem cell source, n (%)	
Peripheral blood	244 (91.7%)
Bone marrow	22 (8.3%)
Donor age, years, median (IQR)	40.0 (31.0–48.0)
Female donors to male recipients, n (%)	
Yes	38 (14.3%)
No	228 (85.7%)
Donor/recipient CMV serostatus, n (%)	
Negative/negative	94 (35.3%)
Negative/positive	57 (21.4%)
Positive/positive	78 (29.3%)
Positive/negative	37 (13.9%)
Graft-versus-host disease prophylaxis, n (%)	
Cyclosporine, MTX	174 (65.4%)
Cyclosporine, MMF	83 (31.2%)
Post-transplant cyclophosphamide, tacrolimus, MMF	9 (3.4%)
Antithymocyte globulin (ATG), n (%)	
Yes	201 (75.6%)
No	65 (24.4%)

*IQR* interquartile range, *ELN* European LeukemiaNet, *allo-HSCT* allogeneic hematopoietic stem cell transplantation, *CMV* cytomegalovirus, *MTX* Methotrexate, *MMF* Mycophenolate mofetil

**Table 2:** Conditioning regimens

Regimens	n (%)
FBM (Fludarabine, BCNU, Melphalan) Fludarabine 5 × 30 mg/m^2^ on five consecutive days (d-8 to d-4), BCNU 2 × 150 mg/m^2^ (d-6, d-5), Melphalan 110 mg/m^2^ on d-3 (age ≥ 55 years) or Melphalan 140 mg/m^2^ on d-3 (age < 55 years)	144 (54.1%)
FTM (Fludarabine, Thiopeta, Melphalan) Fludarabine 5 × 30 mg/m^2^ (d-7 to d-3), Thiopeta 5 mg/kg (d-6), Melphalan 110 mg/m^2^ on d-3 (age ≥ 55 years) or Melphalan 140 mg/m^2^ on d-3 (age < 55 years)	31 (11.6%)
FLAMSA-RIC Treosulfan, Fludarabine FLAMSA regimen (d-12 to d-9): Fludarabine 4 × 30 mg/m^2^, HD-Ara-C 4 × 2000 mg/m^2^, Amsacrine 4 × 100 mg/m^2^. RIC-regimen after 3 days of rest (d-3 to d-5): Fludarabine 3 × 30 mg/m^2^, Treosulfan 3 × 14 g/m^2^ (d-5 to d-3), ATG 10 mg/kg for MRD or 20 mg/kg for MUD, MMRD, MMUD from d -4 to d -2, pDLTs at day + 120 or 30 days after discontinuation of immunosuppression, 1–5 × 10^6^ CD3^+^ cells/kg	20 (7.5%)
FM (Fludarabine, Melphalan) Fludarabine 5 × 30 mg/m^2^ (d-8 to d-4), Melphalan 140 mg/m^2^ (d-4)	18 (6.8%)
Fludarabine, Treosulfan Fludarabine 5 × 30 mg/m^2^ on five consecutive days (d-6 to d-2), Treosulfan 3 × 10 g/m^2^ on three consecutive days (d-4 to d-2)	15 (5.6%)
FLAMSA-RIC Busulfan, Cyclophosphamide FLAMSA regimen (d-12 to d-9): Fludarabine 4 × 30 mg/m^2^, HD-Ara-C 4 × 2000 mg/m^2^, Amsacrine 4 × 100 mg/m^2^. RIC-regimen: Busulfan 4 × 0.8 mg/kg on four consecutive days (d-6 to d-4), Cyclophosphamide (2 × 60 mg/kg for MUD, MMRD and MMUD or 2 × 40 mg/kg for MRD, d-3 to d-2), ATG 10 mg/kg for MRD or 20 mg/kg for MUD, MMRD, MMUD from d-4 to d-2, pDLTs at day + 120 or 30 days after discontinuation of immunosuppression, 1–5 × 10^6^ CD3^+^ cells/kg	8 (3.0%)
Other	30 (11.3%)

*RIC* reduced intensity conditioning, *MRD* matched related donor, *MUD* matched unrelated donor, *MMRD* mismatched related donor, *MMUD* mismatched unrelated donor, *pDLTs* prophylactic donor lymphocyte infusions, *ATG* antithymocyte globulin

### Acute and chronic graft-versus-host disease

The 100-day cumulative incidence of grade II-IV aGVHD was 44.4% [95% CI (38.3, 50.2)], while the 2-year and 5-year cumulative incidences of cGVHD (requiring systemic immunosuppression) were 35.0% [95% CI (29.2, 41.4)] and 36.9% [95% CI (31.1, 42.6)]. Table 3 shows graft-versus-host disease characteristics. The most common sites of cGVHD were skin (77.8%), oral mucosa (67.7%) and eyes (59.6%). Most patients had multi-organ involvement of cGVHD (Table 3). The median time of systemic immunosuppressive therapy of cGVHD was 729.0 days (IQR, 337.5–1715.0) in patients developing cGVHD.

**Table 3:** Chronic graft-versus-host disease

Characteristic	value
Combination of grade II-IV acute and chronic GVHD*, (n = 266)	
No acute GVHD, no chronic GVHD	92 (34.6%)
Acute GVHD, no chronic GVHD	75 (28.2%)
Acute GVHD and chronic GVHD	49 (18.4%)
Chronic GVHD, no acute GVHD	50 (18.8%)
Number of organs affected by chronic GVHD*, median (IQR), (n = 99)	3 (2–4)
Maximum grade of chronic GVHD*, (n = 99)	
Mild	10 (10.1%)
Moderate	42 (42.4%)
Severe	47 (47.5%)
Organs affected by chronic GVHD*, (n = 99)	
Skin	77 (77.8%)
Oral mucosa	67 (67.7%)
Eyes	59 (59.6%)
Liver	29 (29.3%)
Lung	21 (21.2%)
Gastrointestinal tract	16 (16.2%)
Fascia	16 (16.2%)
Genitals	9 (9.1%)
Joints	7 (7.1%)
Central nervous system	4 (4.0%)
Days of systemic immunosuppressive therapy of chronic GVHD, (n = 93)	
Median (IQR)	729.0 (337.5–1715.0)
Mean (95% CI)	1151.2 (95% CI [922.2, 1380.2])

*Chronic GVHD requiring systemic immunosuppressive therapy

### Secondary solid malignancies including precancerous lesions

Table 4 shows all precancerous lesions, atypical nevi (n = 15) and SSMs (n = 42) with the duration of systemic immunosuppression applied for treatment of cGVHD. In summary, 42 SSMs in 32 patients were recorded. The cumulative incidences of any invasive SSMs at 5, 10 and 15 years were 6.0% [95% CI (3.6, 9.3)], 8.6% [95% CI (5.5, 12.4)] and 12.2% [95% CI (8.1, 17.2)], while the cumulative incidences of death (competing risk) at 5, 10 and 15 years were 54.5% [95% CI (48.3, 60.3)], 56.1% [95% CI (49.9, 61.8)] and 58.0% [95% CI (51.3, 64.1)], respectively.

**Table 4:** Secondary solid malignancies, precancerous lesions and atypical nevi after allogeneic hematopoietic stem cell transplantation

Pat.no	Secondary solid malignancies (SSMs), precancerous lesions, atypical nevi	Conditioning regimens	Years from HSCT to SSMs	Age at HSCT	Sex	Grade II–IV acute GVHD	Chronic GVHD requiring systemic immunosuppression, sites of involvement, days of systemic immunosuppression (IS) of cGVHD	Smoker (yes/no)	Death due to SSMs, time from diagnosis of SSMs to death due to SSMs
Invasive squamous cell carcinomas, SCCs (n = 19) in 16 patients									
26	Squamous cell carcinoma, soft palate, G2, pT1 pN0 cM0 L0 V0 Pn0 R0	FBM	2.21	59.8	Male	No	Yes, skin, eyes, liver, oral mucosa, 328 days	Yes	No
352	Cutaneous squamous cell carcinoma, face	FBM	12.54	55.2	Male	Yes	Yes, skin, oral mucosa, gastrointestinal, 5252 days	No	No
86	Squamous cell carcinoma, tongue, G2, pT2 pN0 L0 V0 cM0 R1	FBM	4.39	48.1	Female	No	Yes, oral mucosa, liver, skin, fascia, eyes, 1298 days	No	No
415	Squamous cell carcinoma, cervical carcinoma, pT1b pN1 cM0, HPV pos	Treosulfan, Fludarabine	12.27	43.4	Female	Yes	No	No	No
75	Cutaneous squamous cell carcinoma, face	FBM	11.96	68.2	Male	Yes	Yes, skin, 677 days	No	No
272	Laryngeal cancer, squamous cell, pT1b pN0 cM0, HPV neg	FM	10.89	43.9	Male	No	No	Yes	No
282	Squamous cell carcinoma, tongue, G3, pT1 pN1 R0	Treosulfan, Fludarabine	3.89	69.1	Male	Yes	Yes, eyes, gastrointestinal, oral mucosa, 1863 days	Yes	Yes, 1.65 years
256	Cutaneous squamous cell carcinoma, scalp	FBM	3.15	61.4	Male	Yes	Yes, skin, lung, eyes, 2744 days	No	No
256	Cutaneous squamous cell carcinoma, face	FBM	3.3	61.4	Male	Yes	Yes, skin, lung, eyes, 2744 days	No	No
104	Squamous cell carcinoma, vulva, HPV pos	Thio, Treo, Flud	6.94	57.2	Female	Yes	Yes, skin, oral mucosa, eyes, liver, 254 days	No	No
142	Cutaneous squamous cell carcinoma, face	FBM	10.1	67.3	Male	Yes	Yes, eyes, oral mucosa, lung, 1561 days	No	No
142	Cutaneous squamous cell carcinoma, ear	FBM	10.1	67.3	Male	Yes	Yes, eyes, oral mucosa, lung, 1561 days	Yes	No
142	Cutaneous squamous cell carcinoma, face	FBM	13.1	67.3	Male	Yes	Yes, eyes, oral mucosa, lung, 1561 days	Yes	No
157	Squamous cell cancer, lung, UICC IIIB	FBM	7.90	64.5	Female	No	Yes, skin, eyes, oral mucosa, genitalia, unknown end of IS	Yes	Yes, 0.79 years
31	Esophageal cancer, squamous cell, ypT3 ypN2 L0 V0 R0	FBM	3.2	54.4	Male	Yes	Yes, oral mucosa, eyes, skin, 1355 days	No	Yes, 0.53 years
66	Squamous cell carcinoma, lung, G3, pT2a pN0 (0/21) cM0 L0 V0 R0	FTM	5.8	56.4	Male	No	No	No	No
238	Squamous cell carcinoma, tongue, G2, pT1 pN0 cM0 Pn0 R0 L0	FTM	8.0	40.8	Female	No	Yes, skin, oral mucosa, eyes, lung, joints, 2105 days	yes	No
17	Cutaneous squamous cell carcinoma, face	FBM	1.94	56.1	Male	Yes	Yes, oral mucosa, eyes, lung, 876 days	No	No
63	Cutaneous squamous cell carcinoma, face	FBM	1.95	63.8	Male	Yes	Yes, oral mucosa, eyes, joints, lung, 927 days	No	No
Non-squamous cell carcinomas, non-SCCs (n = 23) in 16 patients									
75	Fibrosarcoma, cheek	FBM	4.19	68.2	Male	Yes	Yes, skin, 677 days	No	No
75	Merkel cell carcinoma, lip, pT2 pN1 cM0	FBM	11.4	68.2	Male	Yes	Yes, skin, 677 days	No	No
104	Malignant melanoma of the skin, back, pT1a pN0 cM0	Thio, Treo, Flud	1.77	57.2	Female	Yes	Yes, skin, oral mucosa, eyes, liver, 254 days	No	No
272	Cutaneous basal cell carcinoma, back	FM	9.92	43.9	Male	No	No	Yes	No
256	Cutaneous basal cell carcinoma, face	FBM	3.31	61.4	Male	Yes	Yes, skin, lung, eyes, 2744 days	No	No
212	Glioblastoma multiforma	FLAMSA-RIC, Bu, Cy	7.21	54.5	Female	No	Yes, oral mucosa, eyes, skin, 1714 days	No	Yes, 1.45 years
263	Gastroesophageal cancer, adenocarcinoma	FLAMSA-RIC, Treo, Flud	2.02	49.2	Female	No	Yes, skin, oral mucosa, 167 days	No	Yes, 0.70 years
397	Cutaneous basal cell carcinoma, nasal bridge	FBM	3.05	65.8	Male	Yes	Yes, oral mucosa, liver, lung, skin, eyes, 1716 days	No	No
309	Cutaneous basal cell carcinoma, nose	FLAMSA-RIC, Treo	3.46	61.4	Male	No	No	No	No
309	Cutaneous basal cell carcinoma, nose	FLAMSA-RIC, Treo	4.6	61.4	Male	No	No	No	No
22	Cutaneous basal cell carcinoma, face	FBM	5.98	60.6	Male	No	No	No	No
403	Follicular thyroid cancer, pT2 pN0 cM0 L0 V1 R0	FTM	4.51	30.6	Female	Yes	No	No	No
66	Conjunctival malignant melanoma, pT1a pN0 cM0 L0 V0 R1	FTM	5.74	56.4	Male	No	No	No	No
66	Adenocarcinoma, lung, pT1b pN2 (8/33) cM0 L1 V0, UICC IIIA	FTM	6.8	56.4	Male	No	No	No	No
21	Cutaneous basal cell carcinoma, face	Treosulfan, Fludarabine	11.33	68.0	Female	Yes	Yes, liver, 796 days	No	No
21	Cutaneous basal cell carcinoma, face	Treosulfan, Fludarabine	11.7	68.0	Female	Yes	Yes, liver, 796 days	No	No
114	Cutaneous basal cell carcinoma, face	FBM	11.53	35.0	Female	Yes	Yes, skin, fascia, eyes, genitalia, liver, gastrointestinal, 685 days	No	No
114	Cutaneous basal cell carcinoma, face	FBM	11.53	35.0	Female	Yes	Yes, skin, fascia, eyes, genitalia, liver, gastrointestinal, 685 days	No	No
236	Cutaneous basal cell carcinoma, face	FBM	2.10	53.5	Male	No	No	No	No
372	Malignant melanoma, arm, pT4b N3 M1b L1 V0 R0	FBM	1.40	54.5	Male	Yes	Yes, skin, 403 days	No	No
436	Cutaneous basal cell carcinoma, cheek	FBM	1.40	64.2	Male	Yes	Yes, liver, 103 days	No	No
436	Cutaneous basal cell carcinoma, face	FBM	1.40	64.2	Male	Yes	Yes, liver, 103 days	No	No
436	Cutaneous basal cell carcinoma, face	FBM	1.40	64.2	Male	Yes	Yes, liver, 103 days	No	No
Precancerous lesions, histologically proven atypical nevi (n = 15) in 11 patients									
7	Atypical nevi, back	FBM	3.1	64.8	Male	No	No	No	No
40	Actinic keratosis, ear	FTM	2.6	44.2	Male	No	No	No	No
62	Atypical nevi, back	FBM	1.3	62.1	Female	Yes	No	No	No
126	Cutaneous carcinoma in situ, arm	FM	14.1	64.0	Male	No	Yes, skin, 5173 days	No	No
126	Cutaneous carcinoma in situ, arm	FM	15.5	64.0	Male	No	Yes, skin, 5173 days	No	No
172	Vulvar intraepithelial neoplasia, VIN III	FLAMSA-RIC, Treo	3.8	43.4	Female	No	Yes, skin, oral mucosa, eyes, fascia, 388 days	No	No
208	Atypical nevi, shoulder	FTM	2.6	56.8	Male	Yes	Yes, skin, 495 days	No	No
238	Cutaneous carcinoma in situ, perineal	FTM	5.7	40.8	Female	No	Yes, skin, oral mucosa, eyes, lung, joints, 2105 days	Yes	No
260	Atypical nevi, hand	FBM	2.9	48.7	Female	Yes	No	No	No
397	Cutaneous carcinoma in situ, face	FBM	8.5	65.8	Male	Yes	Yes, oral mucosa, liver, lung, skin, eyes, 1716 days	No	No
397	Cutaneous carcinoma in situ, face	FBM	8.5	65.8	Male	Yes	Yes, oral mucosa, liver, lung, skin, eyes, 1716 days	No	No
397	Cutaneous carcinoma in situ, face	FBM	8.5	65.8	Male	Yes	Yes, oral mucosa, liver, lung, skin, eyes, 1716 days	No	No
422	Intraepithelial neoplasia, colon	FBM	11.1	55.2	Female	No	No	No	No
429	Cutaneous carcinoma in situ, hand	Treosulfan, Fludarabine	2.4	61.6	Female	No	No	No	No
429	Cutaneous carcinoma in situ, hand	Treosulfan, Fludarabine	2.4	61.6	Female	No	No	No	No

*HPV* human papillomavirus, *FBM* Fludarabine, BCNU, Melphalan, *FTM* Fludarabine, Thiopeta, Melphalan, *FM* Fludarabine, Melphalan, *Treo*, Treosulfan, *Flud* Fludarabine, *BU* Busulfan, *Cy* Cyclophosphamide, *FLAMSA* Fludarabine, Amsacrine, HDAra-C, *RIC* reduced-intensity conditioning, *UICC* Union for International Cancer Control 8th edition

### Secondary squamous cell carcinomas

**Fig. 1:**
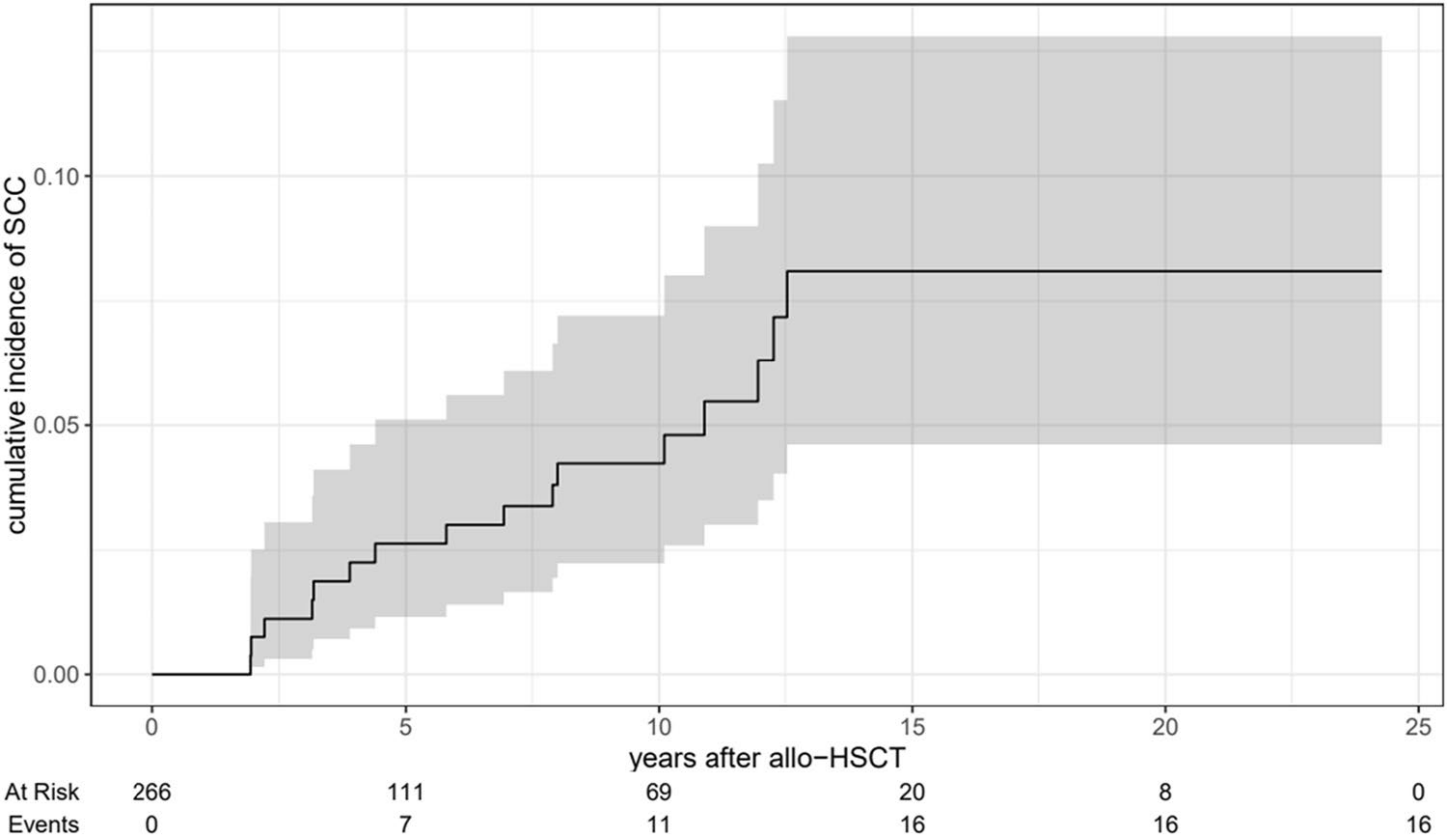
Estimates of the cumulative incidences of invasive squamous cell carcinomas (SCCs) with 95% confidence intervals (competing death)

Nineteen invasive SCCs were recorded in 16 patients (Table 4). Fourteen patients developed one SCC, one patient two SCCs and one patient three SCCs. The most common SCCs were cutaneous SCCs (n = 9) and SCCs of the head and neck region (n = 5). The mean time from allo-HSCT to the development of SCCs was 21.3 years [95% CI (20.0, 22.7)]. Figure 1 shows the estimates of the cumulative incidences of secondary SCCs. The cumulative incidences of SCCs at 5, 10 and 15 years were 2.6% [95% CI (1.2, 5.1)], 4.2% [95% CI (2.2, 7.2)] and 8.1% [95% CI (4.6, 12.8)], respectively (Fig. 1). Within this group of SCCs, the cumulative incidences of cutaneous SCCs at 5, 10 and 15 years were 1.1% [95% CI (0.3, 3.1)], 1.1% [95% CI (0.3, 3.1)] and 3.5% [95% CI (1.3, 7.5)].

### Secondary non-squamous cell carcinomas

**Fig. 2:**
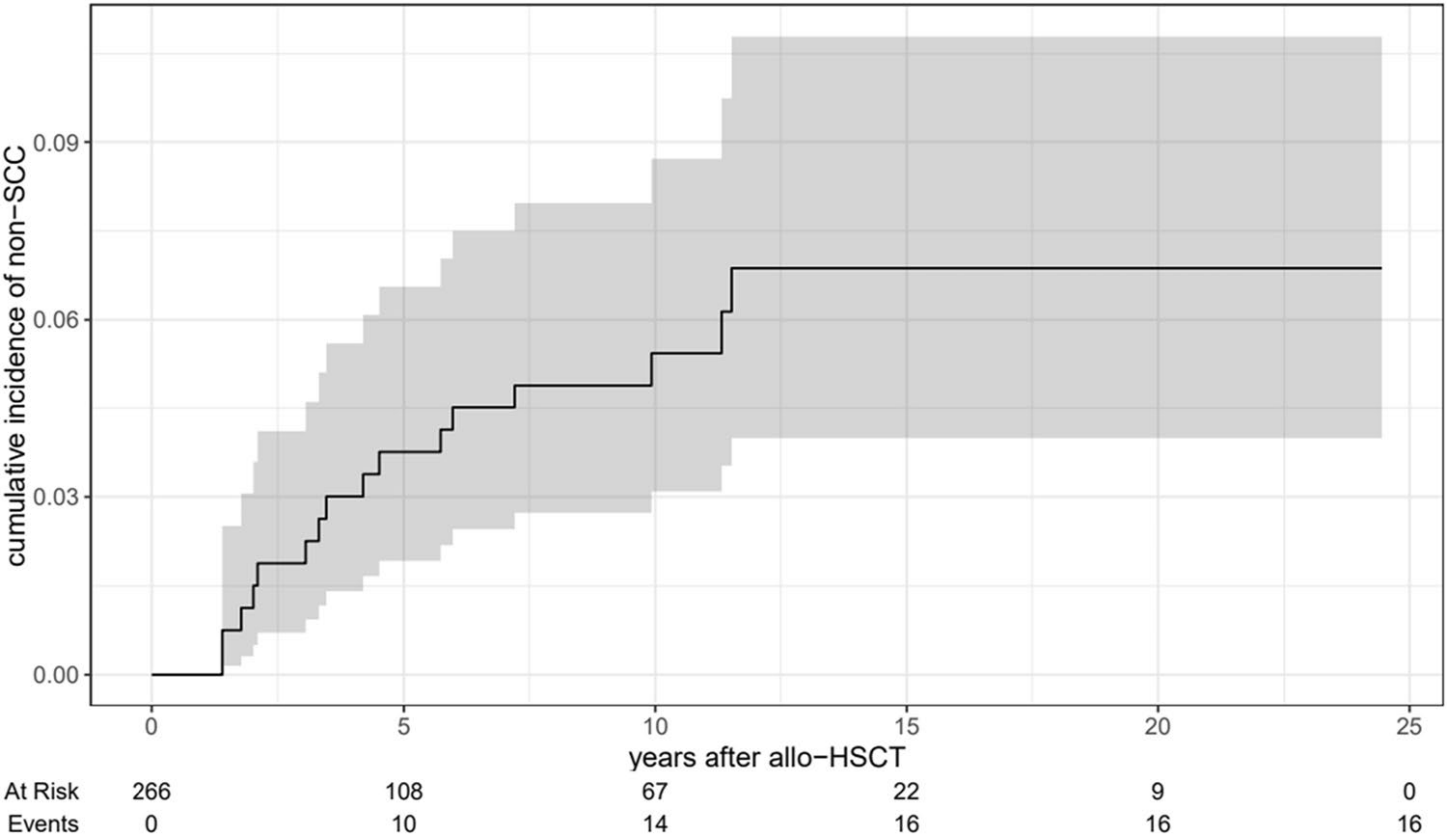
Estimates of the cumulative incidences of secondary non-squamous cell carcinomas (non-SCCs) with 95% confidence intervals (competing death)

In summary, 23 invasive non-SCCs in 16 patients were recorded (Table 4). Ten patients had one non-SCC, five patients had two non-SCCs and one patient had three non-SCCs. The most common cancer types were cutaneous basal cell carcinomas (BCCs, n = 14) and malignant melanomas (n = 3). The mean time from allo-HSCT to the development of invasive non-SCCs was 21.6 years [95% CI (20.3, 23.0)]. The cumulative incidences of non-SCCs at 5, 10 and 15 years were 3.8% [95% CI (1.9, 6.6)], 5.4% [95% CI (3.1, 8.7)] and 6.9% [95% CI (4.0, 10.8)], respectively (Fig. 2). Within this group of non-SCCs, the cumulative incidences of cutaneous BCCs at 5, 10 and 15 years were 1.9% [95% CI (0.7, 4.1)], 2.8% [95% CI (1.2, 5.5)] and 4.3% [95% CI (2.0, 7.9)].

### Precancerous lesions and atypical nevi

Eight cutaneous carcinomas in situ, four histologically proven atypical nevi, one actinic keratosis, one intraepithelial neoplasia of the colon, and one vulvar intraepithelial neoplasia (VIN III) in 11 patients were diagnosed (Table 4). The cumulative incidences of precancerous lesions and atypical nevi at 5, 10 and 15 years were 2.6% [95% CI (1.2, 5.1)], 3.8% [95% CI (2.0, 6.7)] and 4.9% [95% CI (2.4, 8.8)], respectively.

### Multivariate analysis of secondary solid malignancies and pre-transplantation variables

Table 5 depicts the multivariate analysis of pre-transplantation variables and SSMs using multivariable Fine and Gray proportional hazards regression models. The use of ATG was associated with reduced incidences of SCCs [HR, 0.09, 95% CI (0.02, 0.40); *P* = 0.002] compared to patients not receiving ATG. The multivariate analysis found no association of ATG with the incidences of non-SCCs (Table 5).

**Table 5:** Multivariable Fine and Gray proportional hazards regression models of pre-transplantation variables and secondary solid malignancies

	Fine and Gray subdistribution hazard model for					
	Squamous cell carcinomas *competing death*			Non-squamous cell carcinomas *competing death*		
	HR	95% CI	*P*-value	HR	95% CI	*P*-value
Patient age	1.04	0.98, 1.10	0.21	1.01	0.96, 1.06	0.73
Donor type						
Matched sibling donor (reference)						
Matched unrelated donor	4.65	0.90, 24.1	0.067	2.24	0.18, 27.7	0.53
Mismatched unrelated, haploidentical, mismatched related donor	2.19	0.98, 4.90	0.055	0.96	0.26, 3.50	0.95
Donor/recipient CMV status						
Negative/negative (reference)						
Negative/positive	1.77	0.52, 6.05	0.36	1.27	0.25, 6.32	0.77
Positive/positive	0.78	0.17, 3.66	0.75	2.72	0.65, 11.4	0.17
Positive/negative	0.49	0.06, 3.80	0.50	1.55	0.25, 9.75	0.64
Patient sex						
Male (reference)						
Female	0.68	0.21, 2.26	0.53	0.55	0.16, 1.92	0.35
ATG (Antithymocyte globulin)						
No (reference)						
Yes	0.09	0.02, 0.40	0.002	1.45	0.10, 21.1	0.78
Stem cell source						
Peripheral blood (reference)						
Bone marrow	0.30	0.02, 5.45	0.42	0.93	0.04, 22.9	0.96
Female donor to male recipient						
No (reference)						
Yes	1.72	0.38, 7.68	0.48	1.60	0.25, 10.2	0.62
Donor age	1.02	0.97, 1.07	0.49	0.99	0.95, 1.04	0.81
Graft-versus-host prophylaxis						
Cyclosporin A, MTX (reference)						
Cyclosporin A, MMF	0.55	0.16, 1.86	0.33	0.30	0.07, 1.34	0.12
Post-transplant cyclophosphamide, tacrolimus, MMF	0.66	0.07, 6.18	0.71	12.1	1.83, 79.4	0.010
Conditioning regimens						
Melphalan based, RIC (reference)						
Other RIC	0.64	0.16, 2.46	0.51	1.37	0.43, 4.37	0.59

*HR* hazard ratio, *CI* confidence interval, *RIC* reduced-intensity conditioning

### Cause-specific hazard ratios for development of secondary solid malignancies

Tables 6 and 7 show the cause-specific hazard ratios of aGVHD and cGVHD for SSMs after adjustment of patient age (Model 1) and after adjustment of patient age and ATG (Model 2). Patients with grade II-IV aGVHD had significantly increased rates of SCCs after adjusting with patient age and ATG (Table 6), while patients with cGVHD showed only a trend for increased rates of SCCs (Table 7). GVHD variables did not influence the rates of non-SCCs.

**Table 6:** Multivariate analysis of acute graft-versus-host disease for secondary solid malignancies: hazard ratios and 95% confidence intervals from cause-specific hazard models

Variables	Cause-specific hazard regression for					
	Squamous cell carcinomas			Non-squamous cell carcinomas		
	HR	95% CI	*P*-value	HR	95% CI	*P*-value
Model 1						
Grade II–IV acute GVHD	3.18	1.13, 8.93	0.028	2.35	0.86, 6.39	0.094
Patient age	1.05	1.00, 1.11	0.070	1.04	0.99, 1.09	0.2
Model 2						
Grade II–IV acute GVHD	2.83	1.01, 7.93	0.047	2.43	0.89, 6.69	0.084
Patient age	1.08	1.01, 1.14	0.017	1.03	0.98, 1.09	0.2
ATG	0.25	0.09, 0.71	0.010	1.42	0.39, 5.15	0.6

Acute GVHD was analyzed as a time-dependent covariate

*HR* hazard ratio, *CI* confidence intervals, *ATG* antithymocyte globulin

**Table 7:** Multivariate analysis of chronic graft-versus-host disease for secondary solid malignancies: hazard ratios and 95% confidence intervals from cause-specific hazard models

Variables	Cause-specific hazard regression for					
	Squamous cell carcinomas			Non-squamous cell carcinomas		
	HR	95% CI	*P*-value	HR	95% CI	*P*-value
Model 1						
Chronic GVHD requiring systemic immunosuppression	4.75	1.35, 16.8	0.015	1.31	0.49, 3.53	0.6
Patient age	1.06	1.01, 1.12	0.022	1.04	0.99, 1.10	0.090
Model 2						
Chronic GVHD requiring systemic immunosuppression	3.56	0.96, 13.1	0.057	1.37	0.50, 3.76	0.5
Patient age	1.07	1.01, 1.14	0.016	1.04	0.99, 1.10	0.10
ATG	0.34	0.12, 0.96	0.042	1.34	0.37, 4.85	0.7

Chronic GVHD was analyzed as a time-dependent covariate

*HR* hazard ratio, *CI* confidence intervals, *ATG* antithymocyte globulin

## Discussion

This retrospective study analyzed incidences of SSMs including non-melanoma skin cancers after chemotherapy-only conditioning focusing on AML patients and 1st allo-HSCT. In the present study, the overall cumulative incidences of SSMs at 10 and 15 years were 8.6% and 12.2%, similar to recent studies (Martelin et al. 2019; Novitzky-Basso et al. 2020). Novitzky-Basso et al. (2020) revealed overall incidences of SSMs of 19.5% [95% CI (15.9, 23.4)] at 12 years with cutaneous BCCs, cutaneous SCCs and head and neck cancers the most frequent cancer types. Martelin et al. (2019) reported cumulative incidences of SSMs of 13.9% at 15 years (excluding non-melanoma skin cancers). Similar to other studies including non-melanoma skin cancers, cutaneous SCCs and BCCs were the most frequent SSMs in the present study, with cumulative incidences of 3.5% and 4.3% at 15 years. Leisenring et al. (2006) reported 15-year cumulative incidences of cutaneous and mucosal SCCs and BCCs of 2.2% [95% CI (1.7, 2.8)] and 4.0% [95% CI (3.3, 4.8)] in a large cohort of 4,810 patients. Precancerous lesions and atypical nevi were also identified with the help of annual dermatological examinations at the transplant center of Regensburg. The cumulative incidences of precancerous lesions and atypical nevi at 10 and 15 years were 3.8% and 4.9%, respectively. The majority of studies focusing on SSMs did not include non-melanoma skin cancers in the analysis resulting in comparatively low incidences of SSMs. Furthermore, the literature on SSMs shows variability in primary diagnoses (leukemia, lymphoma and aplastic anemia), patient age and conditioning regimens (TBI-based, chemotherapy-only) making a comparison of these results difficult. Acute myeloid leukemia, chronic myeloid leukemia (CML) and acute lymphoblastic leukemia (ALL) seem to have higher risks in comparison to other primary diagnoses (Curtis et al. 1997; Bhatia et al. 2001) contributing to the relatively high cumulative incidences of SSMs of the present study comprising of AML patients.

Chronic GVHD is a main cause of morbidity and mortality after allo-HSCT (Lee et al. 2003). In multivariate analyses, the use of ATG was associated with a lower incidence of secondary SCCs, which may be explained by the reduction in aGVHD and cGVHD (Finke et al. 2009; Kröger et al. 2016). We used a cause-specific hazard model to analyze the effects of aGVHD and cGVHD on the rates of SSMs after adjustment of patient age and ATG. Our results indicate that aGVHD is associated with an increased rate of secondary SCCs after adjusting for patient age and ATG, while cGVHD showed only a trend for an increased rate of SCCs. In summary, the present study confirms the associations between GVHD and secondary SCCs, as indicated by Leisenring et al. (2006) and Curtis et al. (1997). Curtis et al. (1997) concluded that cGVHD is strongly related to the risks for SCCs. This study revealed that patients with cGVHD receiving immunosuppressive therapy for two or more years showed an association with secondary SCCs of the buccal cavity and skin (Curtis et al. 1997). The results of the present study indicate no association of cGVHD with the risks for non-SCC contrary to the results of Leisenring et al. demonstrating a relationship between cGVHD and the risks for non-SCCs (Leisenring et al. 2006). In the present study, four patients developed SCCs of the oral cavity at anatomic sites previously affected by cGVHD, as did the patient with SCC of the esophagus. All patients with malignant melanomas of the skin had a history of cGVHD of the skin. These cases indicate that cGVHD is a relevant risk factor for SSMs of the skin and mucosa (Demarosi et al. 2005). Our results are in line with the review of Demarosi et al. (2005) reporting an elevated risk of SCCs of the oral cavity in patients with cGVHD. Human papillomaviruses (HPV) may further increase the risks of SCCs of the mucosa and skin in patients with systemic immunosuppression (Miller and Johnstone 2001). In the present study, two women with a history of cGVHD developed SCCs of the cervix and vulva both associated with HPV. In summary, the relatively high cumulative incidences of SSMs are not solely based on GVHD but may result from a selection bias, as patients were at relatively old age at the time of allo-HSCT (median age, 55.9 years). This risk-based selection treating older patients with chemotherapy-only conditioning and younger with TBI-based conditioning was defined in institutional guidelines and is in line with recommendations.

The latency period for the development of SSMs is relatively long. Therefore, more SSMs are diagnosed as we obtain longer follow-ups. Literature indicates that GVHD-related SSMs such as SCCs of the skin and oropharynx occur early after allo-HSCT as can be assumed by the cases of the present study. In contrast, the literature indicates that TBI-related SSMs occur with long delay after allo-HSCT (Rizzo et al. 2009). Independent of the conditioning regimens, lifelong screening for SSMs is recommended after allo-HSCT. The screenings for SSMs include skin, thyroid, head and neck and gynecological examinations, as well as assessing symptoms of any kind of SSMs during annual control (Socié and Rizzo 2012). Furthermore, avoidance of additional carcinogenic sources, eg. tobacco, alcohol, and sun exposure are recommended to reduce the risk of SSMs (Socié and Rizzo 2012). This study is limited by its retrospective design and the relatively low number of patients. The primary strength of the present study is the homogeneity of the study population conditioned with non-TBI-based regimens and the details of all SSMs.

## Conclusions

Second solid malignancies occur at any site and histology in patients after allo-HSCT with cutaneous SCCs and BCCs having the highest incidences. Data indicate that aGVHD and cGVHD are risk factors for the development of secondary SCCs after allo-HSCT without association with non-SCCs. Furthermore, the incidences of secondary SCCs seem to be reduced by the use of ATG which results in a reduced incidence of cGVHD. Whether other regimens like post-transplant Cyclophosphamide result in the same reduction of SCCs remains to be shown.

## Data Availability

Datasets generated during the current study are available from the corresponding author on reasonable request.
